# Household financial management behavior and catastrophic health expenditure risk in rural China: the moderating role of health insurance coverage

**DOI:** 10.3389/fpubh.2026.1789846

**Published:** 2026-05-21

**Authors:** Qian Cheng

**Affiliations:** Department of Business, Zibo Polytechnic University, Zibo, Shandong, China

**Keywords:** catastrophic health expenditure, financial management behavior, health insurance, moderating effect, rural households

## Abstract

**Introduction:**

Catastrophic health expenditure (CHE) represents a major cause of poverty among rural households in China. Although health insurance coverage in rural China exceeds 95%, CHE incidence remains high, and existing research has predominantly focused on external protection mechanisms such as health insurance, with insufficient attention to household financial management capacity.

**Methods:**

Drawing on data from 17,156 rural households in the 2019 China Household Finance Survey, this study constructed a financial management behavior index encompassing four dimensions: saving behavior, asset allocation, debt management, and insurance awareness. We employed logistic regression and instrumental variable methods to analyze the impact of financial management behavior on CHE and its interaction with health insurance.

**Results:**

Financial management behavior significantly reduces CHE risk, with saving behavior and insurance awareness demonstrating the strongest protective effects. Health insurance moderates this effect, weakening the protective role by approximately 70%, with stronger substitution among low-capacity households. The protective effect is greater among low-income households, western regions, and older-adult-headed households, exhibiting diminishing marginal utility. Different dimensions show complementary and substitutional relationships: savings’ marginal effect is significantly higher in high-debt households, while debt management’s effect is weaker in high-saving households.

**Discussion:**

These findings suggest that reducing CHE risk requires shifting from singular health insurance expansion toward differentiated “health insurance + financial capacity” strategies, implementing targeted interventions based on household financial capacity levels.

## 1 Introduction

Catastrophic health expenditure occurs when household medical expenditure exceeds a certain threshold of their ability to pay, leading to economic hardship or even poverty. Although health insurance coverage in rural China has exceeded 95%, the incidence of CHE remains far above the 15% control target proposed by the World Health Organization (1, 2), and the marginal effects of supply-side reform measures such as increasing insurance reimbursement rates and expanding coverage are diminishing (3, 4). More critically, facing similar medical expenditure shocks and health insurance conditions, different rural households demonstrate significant variation in their resilience, with some households managing comfortably while others fall into poverty due to illness (5). Recent studies have attempted to introduce the concept of financial literacy to explain this heterogeneity (6, 7), but this approach faces challenges in rural contexts, including urbanized measurement tools, knowledge–behavior gaps, and insufficient data availability (8). Existing financial literacy scales emphasize knowledge about urban financial products such as stocks and funds, which rural households rarely encounter, and possessing financial knowledge does not necessarily translate into sound financial management behavior (9, 10). These challenges suggest that exploring internal protective mechanisms against CHE at the household level may require a new perspective beyond traditional financial literacy frameworks.

Previous research has found that household financial management behaviors including saving behavior, asset allocation, debt management, and insurance participation can influence their capacity to cope with health shocks (11, 12). Recently, the concept of health financial resilience has provided a new theoretical framework for understanding household capacity to respond to health shocks, emphasizing the multidimensional characteristics and dynamic adaptation processes of financial management behavior (13). Savings provide liquidity buffers, diversified asset allocation disperses risk, debt management avoids excessive indebtedness (14, 15), and insurance participation transfers risk. However, existing research predominantly focuses on single dimensions such as saving rates or insurance participation, lacking systematic measurement of household financial management behavior (16). Although some studies have recognized the importance of savings and insurance, they have failed to construct comprehensive indicator systems encompassing multiple dimensions or identify the relative importance of different dimensions. Furthermore, existing measurements are primarily based on knowledge assessment rather than actual behavioral observation, which may lead to systematic bias in rural contexts. More importantly, questions regarding how these four dimensions jointly affect CHE and whether complementary or substitutional relationships exist among them remain inadequately explored.

Regarding the relationship between health insurance and household financial capacity, existing research primarily examines their separate impacts on CHE, with most studies finding that both health insurance and household financial indicators such as savings and assets significantly reduce CHE risk (17, 18). Global systematic review evidence indicates that socioeconomic factors and health system characteristics jointly determine the effectiveness of financial protection indicators, and single-dimension policy interventions often fail to achieve expected effects (19). However, these studies implicitly assume that the two protective mechanisms are independent and simply additive, meaning health insurance provides external protection while household savings provide internal buffers, jointly constructing a safety net (20, 21). Theoretically, however, health insurance as external protection may influence household self-protection. Risk compensation theory suggests that when external protection reduces risk exposure levels, individuals may correspondingly reduce self-protection efforts (22). Resource allocation theory also indicates that health insurance may reduce the necessity of precautionary savings, thereby releasing household financial resources for other uses (23). If such interaction indeed exists between health insurance and financial management behavior, then one-size-fits-all health insurance expansion policies may produce differentiated effects across different households (24). However, existing research has rarely systematically examined the moderating role of health insurance on the effects of financial management behavior, nor quantified the direction and magnitude of this moderating effect.

Regarding heterogeneity research, existing literature commonly finds that the impact of financial capacity on CHE varies across different groups, with higher vulnerability among low-income households, western regions, and older adults. Households headed by older adults, especially those with chronic diseases, face higher CHE risk, and the financial vulnerability of this group deserves special attention (25). These findings are consistent with the law of diminishing marginal utility (26), suggesting that for resource-scarce groups, the marginal value of increasing one unit of financial management capacity is greater. However, heterogeneity analysis in existing research mainly focuses on external groupings such as demographic and economic characteristics like income, region, and age, with less attention to interaction effects among internal dimensions of financial management behavior (27, 28). For example, do households with adequate savings still need strict debt management? Do high-debt households rely more on the buffering role of savings (29)? Does the marginal importance of savings decline for households that have purchased commercial insurance? These questions regarding complementary or substitutional relationships among different financial dimensions not only deepen understanding of the mechanisms of financial management behavior but also have direct policy guidance significance. If substitutional relationships exist among different dimensions, then financial capacity building should not adopt one-size-fits-all strategies but should implement differentiated interventions based on existing household financial structures. However, existing research has not systematically explored heterogeneity at this level.

Based on the above research gaps, this study utilizes data from 17,156 rural households in the 2019 China Household Finance Survey to construct a financial management behavior index encompassing four dimensions—saving behavior, asset allocation, debt management, and insurance awareness—to examine its impact on CHE, the moderating role of health insurance, and complementary-substitutional relationships among different financial dimensions. Based on theoretical analysis and existing literature, we propose the following hypotheses: financial management behavior significantly reduces CHE risk, and the protective effects of the four dimensions differ, with saving behavior and insurance awareness playing more critical roles (Hypothesis 1); health insurance moderates the effect of financial management behavior, weakening its protective effect, and this moderating role is more significant among households with lower financial capacity (Hypothesis 2); complementary or substitutional relationships exist among different dimensions of financial management behavior, with the marginal importance of debt management declining in high-saving households and the marginal importance of savings rising in high-debt households (Hypothesis 3). This study holds theoretical value for understanding the mechanisms of household financial capacity in health risk management and practical significance for designing differentiated medical security policies and household financial capacity building programs, helping to address the policy puzzle of high health insurance coverage coexisting with high CHE incidence from the demand-side perspective.

## 2 Methods

### 2.1 Data source and applicability

This study draws on data from the 2019 China Household Finance Survey conducted by the Survey and Research Center for China Household Finance at Southwestern University of Finance and Economics. The survey employed a stratified three-stage random sampling method, covering 29 provinces, autonomous regions, and municipalities, 343 districts and counties, and 1,360 villages and neighborhood committees, with a sample size of 34,643 households. The selection of the China Household Finance Survey database is primarily based on three advantages: First, it provides the most comprehensive measurement of medical expenditure, including not only traditional outpatient and inpatient costs but also modern health consumption expenditures such as exercise, fitness, and health supplements, enabling more accurate reflection of households’ true health economic burden; Second, it contains detailed household financial information including savings, assets, liabilities, and insurance, providing a data foundation for constructing a multidimensional financial management behavior index; Third, the large sample size and national representativeness make it suitable for large-sample empirical analysis and heterogeneity testing.

### 2.2 Sample selection

Starting from the total sample of 34,643 households in the 2019 China Household Finance Survey, we conducted the following sequential screening: First, we selected 20,125 rural registered households, accounting for 58.1% of the total sample; Second, we excluded 1,891 samples with missing values in core variables including medical expenditure, total household consumption, food expenditure, income, assets, and liabilities; Third, we winsorized medical expenditure and income variables at the 1st and 99th percentiles and excluded 1,078 extreme values, ultimately obtaining an analytical sample of 17,156 households, accounting for 49.5% of the total sample. To assess potential selection bias introduced by listwise deletion, we compared the excluded sample (*n* = 1,891) with the analytical sample (*n* = 17,156) on key demographic and economic characteristics including age, income, region, and chronic disease status. No statistically significant differences were found between the two groups, suggesting that missing data occurred largely at random and that listwise deletion is unlikely to introduce systematic bias.

### 2.3 Variable measurement

#### 2.3.1 Core independent variable: financial management behavior index

This study constructed a comprehensive financial management behavior index encompassing four dimensions, with a total score of 100 points and a maximum of 25 points per dimension. Measurement indicators and theoretical foundations for each dimension are shown in Table 1.

**Table 1:** Components of financial management behavior index.

Dimension	Measurement indicators	Variable type	Theoretical foundation	Mechanism of action
Saving behavior (25 points)	Whether has deposits	Binary	Precautionary saving theory	Liquidity buffer
	Saving rate level	Continuous		Cope with high-frequency low-loss risks
	Saving stability	Ordinal		
Asset allocation (25 points)	Number of financial asset types	Count	Portfolio theory	Risk diversification
	Whether holds risky assets	Binary		Reduce wealth volatility exposure
	Asset allocation diversification	Ordinal		
Debt management (25 points)	Asset-liability ratio level	Continuous	Credit constraint theory	Debt constraint
	Whether has informal loans	Binary		Avoid debt traps
	Debt structure rationality	Ordinal		
Insurance awareness (25 points)	Whether purchased commercial insurance	Binary	Risk management theory	Risk transfer
	Insurance expenditure ratio	Continuous		Cope with low-frequency high-loss risks
	Insurance coverage scope	Ordinal		

Binary indicators were coded as 0 or 1; continuous indicators were divided into quartiles and scored 0–3; ordinal and count indicators were scored 0–3 based on their ordered categories. Indicator scores within each dimension were summed with equal weights and linearly rescaled to a 0–25 range. “Insurance Awareness” in this study refers to households’ actual insurance participation behaviors, operationalized through three objective indicators: commercial insurance ownership, insurance expenditure ratio, and insurance coverage scope, rather than subjective knowledge or attitudinal measures.

This behavior-oriented measurement system has three advantages over traditional financial literacy scales: First, it avoids urban bias by not assessing knowledge about financial products such as stocks and funds that rural households rarely encounter; Second, it overcomes the knowledge–behavior gap by measuring actual behaviors rather than abstract knowledge levels; Third, it enhances policy actionability, as each dimension corresponds to clear policy intervention points.

#### 2.3.2 Dependent variable, moderating variable, and control variables

Main variable definitions are shown in Table 2.

**Table 2:** Variable definitions and measurements.

Variable type	Variable name	Definition and measurement
Dependent variable		
	Catastrophic Health Expenditure (CHE)	WHO ability-to-pay method: out-of-pocket medical expenditure/ability to pay > threshold, coded as 1, otherwise 0 Ability to pay = total household consumption expenditure–food expenditure Out-of-pocket medical expenditure = total medical expenditure–health insurance reimbursement Primary thresholds: 10 and 40%
Moderating variable		
	Health Insurance Coverage	Participated in New Rural Cooperative Medical Scheme or Urban–Rural Resident Medical Insurance = 1, otherwise = 0
Control variables		
Household economic characteristics	Per capita income	Total household income/household size, logarithm
	Per capita assets	Total household assets/household size, logarithm
Population structure characteristics	Household size	Number of household members (persons)
	Dependency ratio	Non-working-age population/working-age population (reflects household non-labor burden)
	Has chronic disease patient	At least one household member has chronic disease = 1, otherwise = 0
Household head characteristics	Age	Household head age (years)
	Years of education	Household head years of education (years)
	Gender	Male = 1, Female = 0
	Marital status	Married = 1, Unmarried/Divorced/Widowed = 0
Fixed effects	Provincial fixed effects	Control for provincial dummy variables, eliminate unobserved heterogeneity across regions

The dependency ratio reflects household non-labor burden and can more comprehensively reflect the dual burden of children and older adults compared to the proportion of older adults alone.

### 2.4 Model specification and causal identification

#### 2.4.1 Baseline model

We employed a logit model to estimate the impact of financial management behavior on CHE:


$$P(\mathrm{CH}E_{i}=1|X_{i})=\Lambda (\beta_{0}+\beta_{1}\mathrm{FM}B_{i}+\beta_{2}\text{Control}s_{i}+\gamma_{p})$$ (1)

where $\mathrm{CH}E_{i}$ indicates whether catastrophic health expenditure occurred, $\mathrm{FM}B_{i}$ represents the financial management behavior index, $\text{Control}s_{i}$ denotes control variables, $\gamma_{p}$ represents provincial fixed effects, and Λ(⋅) is the logistic cumulative distribution function, $\beta_{0}$ is the intercept, $\beta_{1}$ is the core parameter reflecting the marginal effect of financial management behavior, and $\beta_{2}$ is the coefficient vector of control variables. The baseline specification is presented in Equation 1.

We adopted a strategy of progressively adding control variables: Model 1 includes only $\mathrm{FM}B_{i}$ and provincial fixed effects, Model 2 adds household economic characteristics, and Model 3 adds population structure and household head characteristics. Additionally, we decomposed the index into four dimensions for separate regression to examine relative importance.

#### 2.4.2 Instrumental variable method

Financial management behavior may suffer from endogeneity: households anticipating high medical risk may increase savings in advance (reverse causality), and unobserved factors such as time preference and risk preference may simultaneously affect financial behavior and CHE (omitted variables). We employed two-stage least squares (2SLS), selecting village-level financial service accessibility as instrumental variables, including whether there is a bank branch in the village and whether there is an insurance service point in the village.

The instrumental variables satisfy relevance and exogeneity conditions. Relevance: village-level financial service accessibility reduces household transaction costs for obtaining financial services, directly affecting their saving, asset allocation, debt management, and insurance participation behaviors. Exogeneity: village-level financial service layout is determined by financial institutions’ business decisions and government planning, unrelated to individual household health status and medical expenditure. We report the first-stage F-statistic to test for weak instrument problems (*F* > 10 indicates sufficient instrument strength) and the Sargan over-identification test to examine instrument exogeneity (*p* > 0.1 accepts the exogeneity hypothesis).

#### 2.4.3 Moderating effect model

We added interaction terms to the baseline model to test the moderating role of health insurance:


$$P(\mathrm{CH}E_{i}=1|X_{i})=\Lambda (\begin{array}{l} \beta_{0}+\beta_{1}\mathrm{FM}B_{i}+\beta_{2}\text{Insuranc}e_{i}+\beta_{3}\mathrm{FM}B_{i}\\ \times \text{Insuranc}e_{i}+\beta_{4}\text{Control}s_{i}+\gamma_{p} \end{array})$$ (2)

where $\text{Insuranc}e_{i}$ is the health insurance coverage dummy variable, $\beta_{3}$ is the interaction term coefficient reflecting the moderating effect: if significantly positive, it indicates health insurance weakens the protective effect of financial management behavior; if significantly negative, it indicates health insurance strengthens the protective effect. $\beta_{4}$ is the coefficient vector of control variables. The moderating effect specification is presented in Equation 2. We further conducted grouped regressions by insurance status to compare differences in financial management behavior coefficients between the two groups.

### 2.5 Robustness tests and heterogeneity analysis

We employed the following methods to test result sensitivity to model specifications. Replacing dependent variable thresholds: We redefined CHE using different thresholds such as 15, 20, and 25% to test baseline result sensitivity to threshold selection. Replacing estimation models: We re-estimated using Probit models and linear probability models (LPM) instead of logit models to test result sensitivity to model specification. Excluding extreme values: We excluded samples in the top and bottom 5% of the financial management behavior index to test whether extreme values drive main results.

We examined heterogeneity from three levels. Panel A: Financial dimension interaction effects: We examined the effect of the debt management dimension separately in high and low saving households, examined the effect of the saving behavior dimension separately in high and low debt households, and examined the effect of the saving behavior dimension separately in households with and without commercial insurance, to identify complementary or substitutional relationships among different dimensions of financial management behavior. Grouping criteria: high-saving households are those with saving behavior scores above the median, high-debt households are those with asset-liability ratios above 50%, low-debt households are those with asset-liability ratios below 30%, and moderate-debt households (asset-liability ratio 30–50%, approximately 2,000 households) are excluded from this analysis to highlight the contrast between extreme groups. Panel B: Financial capacity heterogeneity in insurance moderation: We divided the sample into three groups by financial management behavior index: low financial capacity (<30 points), moderate financial capacity (30–60 points), and high financial capacity (>60 points), and separately estimated financial management behavior coefficients for insured and uninsured households in each group, calculating insurance substitution rate = (uninsured coefficient - insured coefficient) / absolute value of uninsured coefficient, to examine how the substitution effect of health insurance varies with household financial capacity. Panel C: Demographic and economic characteristic heterogeneity: We conducted grouped regressions by income quintile, region (eastern, central, western), and household head age (≤45 years, 46–60 years, ≥61 years) to examine differences in the protective effect of financial management behavior across different groups. All heterogeneity analyses control for complete control variables and provincial fixed effects.

## 3 Results

### 3.1 Descriptive statistics

Table 3 presents descriptive statistics, between-group differences, and the association trend between financial management behavior and CHE for main variables. Panel A reports comparisons of the full sample and groups divided by CHE status. Households experiencing CHE are significantly weaker in financial management behavior than households not experiencing CHE. Specifically, the average financial management behavior index for households experiencing CHE is 36.2 points, significantly lower than 52.1 points for households not experiencing CHE, with a difference of 15.9 points significant at the 1% level. All four dimensions show similar patterns, with households experiencing CHE scoring significantly lower in saving behavior, asset allocation, debt management, and insurance awareness than households not experiencing CHE, with the differences most pronounced in saving behavior and insurance awareness. Regarding health insurance coverage, the difference between the two groups is relatively small but still significant, with CHE households having an insurance coverage rate of 94.2%, slightly lower than 95.2% for non-CHE households.

**Table 3:** Descriptive statistics, between-group differences, and association trends.

Panel A: descriptive statistics and between-group differences for main variables				
Variable	Full sample (*N* = 17156)	CHE = 0 (*N* = 9899)	CHE = 1 (*N* = 7257)	Difference (t/χ^2^)
Financial management behavior				
Financial management behavior index	45.3 (18.7)	52.1 (17.4)	36.2 (17.3)	15.9***
Saving behavior score	12.3 (6.8)	14.2 (6.5)	9.8 (6.4)	4.4***
Asset allocation score	8.5 (5.2)	9.3 (5.4)	7.4 (4.8)	1.9***
Debt management score	15.7 (7.3)	17.5 (6.8)	13.2 (7.2)	4.3***
Insurance awareness score	8.8 (6.1)	11.1 (6.3)	5.7 (4.9)	5.4***
Moderating variable				
Urban–rural resident medical insurance	0.948	0.952	0.942	0.010**
Control variables				
Household head age (years)	56.3 (12.8)	54.8 (12.4)	58.4 (13.0)	−3.6***
Years of education (years)	7.2 (3.5)	7.8 (3.6)	6.4 (3.2)	1.4***
Household size (persons)	3.8 (1.6)	3.9 (1.6)	3.7 (1.5)	0.2***
Dependency ratio	0.52 (0.41)	0.49 (0.39)	0.56 (0.43)	−0.07***
Has chronic disease patient	0.457	0.382	0.561	−0.179***
Per capita annual income (10,000 yuan)	2.43 (2.16)	2.85 (2.31)	1.87 (1.85)	0.98***
Per capita assets (10,000 yuan)	35.2 (48.3)	39.7 (51.2)	29.1 (43.8)	10.6***

Panel B: association trend between financial management behavior and catastrophic health expenditure				
Financial behavior quartile	Sample size	Financial behavior index mean (SD)	CHE incidence 10% threshold	CHE incidence 40% threshold
Q1 (Lowest quartile)	4289	23.1 (6.5)	38.5%	15.4%
Q2 (Second quartile)	4289	38.5 (4.2)	31.2%	12.5%
Q3 (Third quartile)	4289	52.2 (4.8)	25.3%	10.1%
Q4 (Highest quartile)	4289	68.7 (7.6)	19.8%	7.9%
Q1 vs. Q4 difference	-	−45.6***	18.7pp***	7.5pp***
Cochran-Armitage trend test	-	-	χ^2^ = 528.4***	χ^2^ = 782.1***

***p < 0.01, **p < 0.05, *p < 0.1; Parentheses in Panel A contain standard deviations; CHE grouping uses 40% threshold; Difference column represents mean difference of CHE = 0 group minus CHE = 1 group (negative values indicate CHE = 0 group has lower values); Between-group differences for continuous variables use independent samples t-tests, binary variables use chi-square tests; In Panel B, pp indicates percentage points, quartiles are divided equally by financial management behavior index to ensure equal sample sizes in each group, Cochran-Armitage trend test examines whether CHE incidence shows a monotonic decreasing trend with increasing financial management behavior.

For control variables, households experiencing CHE exhibit higher health risks and weaker socioeconomic status. The average age of household heads experiencing CHE is 58.4 years, significantly higher than 54.8 years for non-CHE households; years of education average 6.4 years, significantly lower than 7.8 years for non-CHE households; chronic disease prevalence is 56.1%, significantly higher than 38.2% for non-CHE households; per capita annual income is 18,700 yuan, significantly lower than 28,500 yuan for non-CHE households. These significant between-group differences indicate that households with weaker financial management behavior, lower socioeconomic status, and higher health risks are more likely to suffer CHE shocks.

Panel B further demonstrates the association trend between financial management behavior and CHE. As financial management behavior levels increase, CHE incidence shows a monotonic decreasing trend. At the 10% threshold, CHE incidence in the lowest quartile group is 38.5%, compared to 19.8% in the highest quartile group, a difference of 18.7 percentage points; at the 40% threshold, incidence in the lowest quartile group is 55.3%, compared to 28.7% in the highest quartile group, a difference of 26.6 percentage points. The Cochran-Armitage trend test is significant, confirming the negative association between financial management behavior and CHE. This decreasing trend remains consistent at both thresholds, and the difference expands further as the threshold increases, suggesting that financial management behavior has a stronger protective effect against more severe medical economic burdens.

### 3.2 Baseline regression results

Table 4 reports the impact of financial management behavior on CHE. The first three columns show result robustness using a strategy of progressively adding control variables. The coefficient of the financial management behavior index remains robust across the three models, from −0.087 in Model 1 to −0.082 in Model 3, with minimal variation and consistently significant at the 1% level, with an odds ratio of 0.917 indicating an 8.3% reduction in odds. After controlling for all confounding factors, the negative impact of financial management behavior on CHE remains robust. To focus on the role of financial management behavior and demonstrate its independent effect, the first three columns do not yet control for the health insurance variable; the moderating role of health insurance will be specifically examined in Section 3.3.

**Table 4:** Impact of financial management behavior on catastrophic health expenditure.

Variable	Model 1	Model 2	Model 3	Saving behavior	Asset allocation	Debt management	Insurance awareness	Four dimensions simultaneously
Financial management behavior index	−0.087***	−0.083***	−0.082***	-	-	-	-	-
	(0.004)	(0.004)	(0.004)					
Saving behavior dimension	-	-	-	−0.118***	-	-	-	−0.102***
				(0.018)				(0.021)
Asset allocation dimension	-	-	-	-	−0.062**	-	-	−0.054*
					(0.020)			(0.023)
Debt management dimension	-	-	-	-	-	−0.089***	-	−0.077**
						(0.016)		(0.019)
Insurance awareness dimension	-	-	-	-	-	-	−0.106***	−0.095***
							(0.019)	(0.022)
Household per capita income		−0.045***	−0.038***	Yes	Yes	Yes	Yes	Yes
Household per capita assets		−0.022***	−0.018**	Yes	Yes	Yes	Yes	Yes
Population structure variables			Yes	Yes	Yes	Yes	Yes	Yes
Provincial fixed effects	Yes	Yes	Yes	Yes	Yes	Yes	Yes	Yes
Sample size	17156	17156	17156	17156	17156	17156	17156	17156
Pseudo R^2^	0.082	0.128	0.145	0.148	0.142	0.152	0.145	0.162
Odds ratio	-	-	0.917***	0.889*	0.940*	0.915**	0.899*	-

***p < 0.01, **p < 0.05, *p < 0.1; Parentheses contain standard errors; Population structure variables include household head age, years of education, household size, dependency ratio, and presence of chronic disease patient; Odds ratio is exp(coefficient); The first three columns do not control for health insurance variable to demonstrate the independent effect of financial management behavior; moderating role of health insurance is shown in Table 5.

The last four columns of Table 4 report separate regression results for the four dimensions of financial management behavior. All four dimensions have significant negative impacts on CHE, but protective effects differ. Saving behavior has the strongest effect with an odds ratio of 0.889 (exp(−0.118)), insurance awareness is second strongest with an odds ratio of 0.899 (exp(−0.106)), debt management has an odds ratio of 0.915 (exp(−0.089)), and asset allocation has a relatively weaker effect with an odds ratio of 0.940 (exp(−0.062)). When all four dimensions are included in the model simultaneously, coefficients for each dimension remain significant, indicating that different dimensions of financial management behavior have independent and complementary effects in reducing CHE risk. This result supports research Hypothesis 2, that the four dimensions of financial management behavior have different impacts on CHE, with saving behavior and insurance awareness being most significant.

### 3.3 Moderating effect analysis

Table 5 reports the moderating role of health insurance on the effect of financial management behavior. The interaction term model shows that health insurance significantly weakens the protective effect of financial management behavior, with the interaction term coefficient significantly positive at 0.067, indicating that health insurance reduces the protective effect of financial management behavior by approximately 70.5%. In uninsured households, a 10-point increase in the financial management behavior index reduces the log-odds of CHE by 0.95 (coefficient −0.095), while this effect weakens to 0.28 (coefficient −0.028) in insured households. In terms of predicted probabilities, when control variables are set at their means and the index increases from 20 to 80 points, CHE probability in uninsured households decreases from 45.2 to 15.4% (a reduction of 29.8 percentage points), while in insured households it decreases from 28.1 to 12.8% (a reduction of 15.3 percentage points), with health insurance playing a stronger protective role in low financial capacity households.

**Table 5:** Moderating role of health insurance.

Variable	Without interaction	Interaction model	Uninsured group	Insured group
Financial management behavior index	−0.082*** (0.004)	−0.095*** (0.006)	−0.095*** (0.014)	−0.028** (0.011)
Urban–rural resident medical insurance	−0.218*** (0.042)	−0.245** (0.082)	-	-
Behavior index × Medical insurance	-	0.067** (0.026)	-	-
Control variables	Yes	Yes	Yes	Yes
Provincial fixed effects	Yes	Yes	Yes	Yes
Sample size	17156	17156	892	16264
Pseudo R^2^	0.156	0.185	0.198	0.172
Moderating effect quantification				
Insurance weakens behavior effect	-	70.5%	-	-
Between-group coefficient difference test	-	*p* = 0.008	-	-
Predicted probability example (Control Variables = Mean)				
CHE probability at index 20 points	-	-	45.2%	28.1%
CHE probability at index 80 points	-	-	15.4%	12.8%
Risk reduction from 60-point increase	-	-	29.8pp	15.3pp
Insurance risk reduction proportion (at low financial capacity)	-	-	37.8%	-

***p < 0.01, **p < 0.05; Parentheses contain standard errors; pp indicates percentage points; Control variables include all variables in Table 2; Urban–rural resident medical insurance includes New Rural Cooperative Medical Scheme; In predicted probability examples, “risk reduction proportion” refers to the absolute risk reduced by insurance as a proportion of risk without insurance.

Grouped regression results further confirm the moderating effect. The marginal effect in the uninsured group is 3.4 times that in the insured group, with significant between-group coefficient differences (*p* = 0.008). The predicted probability examples reported in Table 5 show that when the financial management behavior index is 20 points, health insurance reduces risk from 45.2 to 28.1% (a reduction of 17.1 percentage points), with risk reduction accounting for 37.8%; when the index increases to 80 points, the marginal protective effect of health insurance substantially weakens (from 28.1 to 12.8%, only a 15.3 percentage point reduction). These results support research Hypotheses 3 and 4, that health insurance coverage reduces CHE incidence risk but its protective effect varies across different households, providing critical protection for households with poor financial management behavior while showing diminishing marginal protective effects for households with good financial management behavior, with health insurance and financial management behavior having a partial substitution relationship.

### 3.4 Robustness tests

Table 6 reports robustness test results, with all tests supporting baseline regression conclusions. Threshold replacement tests show that when using different thresholds such as 15, 20, and 25%, the financial management behavior index coefficient remains consistently significantly negative with absolute values varying little between 0.074 and 0.082, indicating baseline results are insensitive to threshold selection. Model replacement tests show that using Probit models and linear probability models yields consistent conclusions. Extreme value exclusion tests show that after removing the top and bottom 5% of the financial management behavior index, the coefficient absolute value is slightly larger than the baseline result, suggesting extreme values may underestimate the true effect.

**Table 6:** Robustness test results.

Test method	CHE threshold/model	Behavior index coefficient	*p*-value	Sample size
Baseline result	CHE1 (10%)	−0.082***	<0.001	17156
Replace threshold	CHE (15%)	−0.082***	<0.001	17156
Replace threshold	CHE (20%)	−0.078***	<0.001	17156
Replace threshold	CHE (25%)	−0.074***	<0.001	17156
Replace model	Probit	−0.053***	<0.001	17156
Replace model	LPM	−0.029***	<0.001	17156
Exclude extreme values	Remove top/bottom 5%	−0.091***	<0.001	15440
Instrumental variable method				
IV first stage: Bank in village	On financial behavior index	3.82***	<0.001	17156
IV first stage: Insurance point in village	On financial behavior index	2.47***	<0.001	17156
IV first stage F-statistic	-	152.7	-	-
IV second stage	2SLS estimation	−0.112***	<0.001	17156
Sargan over-identification test	χ^2^ statistic	0.91	0.342	-

***p < 0.01; Except LPM which shows marginal effects, others are logit coefficients; First stage F-statistic >10 indicates no weak instrument problem; Sargan test *p* > 0.1 accepts instrument exogeneity hypothesis.

Instrumental variable test results show that the first-stage F-statistic is 152.7, far exceeding the critical value of 10, indicating no weak instrument problem, and the Sargan over-identification test *p*-value is 0.342, accepting the exogeneity hypothesis. Second-stage regression results show that the financial management behavior index coefficient is −0.112 and significant at the 1% level, with an absolute value larger than the baseline regression coefficient of −0.082, further confirming the causal effect of financial management behavior on CHE. The larger absolute value of the 2SLS estimate relative to the OLS baseline suggests that the baseline regression may suffer from attenuation bias, likely due to classical measurement error in the self-reported financial management behavior index. The instrumental variable approach circumvents this issue by exploiting exogenous variation in village-level financial service accessibility, and the 2SLS estimate may therefore more closely approximate the true protective effect of financial management behavior.

### 3.5 Heterogeneity analysis

Table 7 reports heterogeneity analysis results, deepening understanding of the mechanisms of financial management behavior from three levels. Panel A results show that significant complementary and substitutional relationships exist among different dimensions of financial management behavior. Savings and debt management exhibit a substitutional relationship, with the protective effect of debt management in low-saving households being 2.5 times that in high-saving households, with significant between-group differences (*p* = 0.012). Savings and debt exhibit a complementary relationship, with the protective effect of savings in high-debt households being 2.2 times that in low-debt households, with significant between-group differences (*p* = 0.008). Savings and insurance also have some substitutional relationship, with the protective effect of savings in households without commercial insurance being 1.7 times that in households with commercial insurance, with marginally significant between-group differences (*p* = 0.055).

**Table 7:** Heterogeneity analysis: financial dimension interactions, insurance moderation heterogeneity, and conventional characteristic heterogeneity.

Panel A: interaction effects among financial management behavior dimensions							
Grouping dimension	Grouping criteria	Sample size	Dimension effect examined	Coefficient	Standard error	*p*-value	Between-group difference
Saving level	High saving (>median)	8578	Debt management	−0.015*	0.008	0.061	*p* = 0.012**
	Low saving (≤median)	8578	Debt management	−0.038***	0.011	<0.001	
Debt level	High debt (>50%)	3289	Saving behavior	−0.048***	0.015	0.001	*p* = 0.008***
	Low debt (≤30%)	11867	Saving behavior	−0.022**	0.009	0.014	
Insurance status	Has commercial insurance	2847	Saving behavior	−0.025**	0.010	0.012	*p* = 0.055*
	No commercial insurance	14309	Saving behavior	−0.042***	0.008	<0.001	

Panel B: financial capacity heterogeneity in insurance moderation					
Financial management capacity	Sample size	Insured coefficient	Uninsured coefficient	Insurance substitution rate	Between-group difference
Low financial capacity (<30 points)	2847	−0.018*	−0.165***	89.1%	Reference group
		(0.009)	(0.038)		
Moderate financial capacity (30–60 points)	10562	−0.035***	−0.082***	57.3%	*p* = 0.003***
		(0.008)	(0.019)		
High financial capacity (>60 points)	3747	−0.048***	−0.068***	29.4%	*p* < 0.001***
		(0.012)	(0.021)		

Panel C: conventional demographic and economic characteristic heterogeneity						
Grouping dimension	Grouping category	Sample size	Financial behavior coefficient	Standard error	*p*-value	Between-group difference
Income level	Low income (Q1)	3431	−0.135***	0.017	<0.001	Q1 vs. Q5:
	High income (Q5)	3432	−0.041*	0.015	0.082	*p* < 0.001***
Region	Eastern	5717	−0.068***	0.014	<0.001	Western vs. Eastern:
	Western	6293	−0.112***	0.018	<0.001	*p* = 0.008***
Age	≤45 years	3089	−0.071**	0.021	0.008	≥61 vs. ≤ 45:
	≥61 years	6543	−0.102***	0.017	<0.001	*p* = 0.018**

***p < 0.01, **p < 0.05, *p < 0.1; Parentheses contain standard errors; Coefficients in Panel A represent the impact of a 10-point increase in the examined dimension on CHE within that group, controlling for the other three dimensions and all control variables; Debt level grouping excludes moderate-debt households with asset-liability ratios between 30 and 50% (approximately 2,000 households), comparing only high-debt and low-debt extreme groups; Insurance substitution rate in Panel B = (uninsured coefficient - insured coefficient) / absolute value of uninsured coefficient, this indicator is calculated based on regression coefficients and differs from the “risk reduction proportion” calculated based on predicted probabilities in Table 5; Panel C shows only extreme group comparisons: for income quintiles only Q1 and Q5 are listed, for region tri-classification only eastern and western are listed, for age tri-grouping only ≤45 years and ≥61 years are listed.

Panel B results show that the moderating role of health insurance exhibits significant differences across households with different financial management capacities, deepening the overall moderating effect findings from Section 3.3. In low financial capacity households, the insurance substitution rate reaches 89.1%, indicating that health insurance almost completely substitutes for the protective role of financial management behavior in low financial capacity households. In moderate financial capacity households, the insurance substitution rate is 57.3%, significantly lower than the low financial capacity group (*p* = 0.003). In high financial capacity households, the insurance substitution rate is only 29.4%, significantly lower than the moderate financial capacity group (*p* < 0.001). As household financial management capacity increases, the substitution role of health insurance shows a clear decreasing trend.

Panel C results show that the protective effect of financial management behavior exhibits significant differences across groups with different demographic and economic characteristics. The protective effect in the low-income group is 3.3 times that in the high-income group, with significant between-group differences (*p* < 0.001). The protective effect in western regions is 1.6 times that in eastern regions, with significant between-group differences (*p* = 0.008). The protective effect in older adults household head families is 1.4 times that in young and middle-aged families, with significant between-group differences (*p* = 0.018). These heterogeneity results indicate that financial management behavior plays a stronger protective role among groups with scarce resources, relatively weak medical security, and higher health risks.

## 4 Discussion

This study finds that financial management behavior significantly reduces rural household CHE risk through three mechanisms, with saving behavior and insurance awareness demonstrating the strongest protective effects among the four dimensions. The liquidity buffer mechanism emphasizes that adequate savings and liquid asset allocation can provide immediate financial support when medical expenditure suddenly increases (6). The importance of this mechanism may extend beyond solving ability-to-pay problems. We hypothesize that adequate savings could also reduce household health anxiety: when households know they have sufficient reserves, they may be more likely to seek treatment in the early stages of illness, potentially avoiding the vicious cycle where delaying minor illnesses leads to worsening conditions and greater expenditure. This hypothesized behavioral pathway warrants direct empirical testing in future research. The instrumental variable method’s estimated coefficient absolute value is larger than baseline regression, suggesting this causal effect may be underestimated. The risk diversification mechanism refers to diversified asset allocation reducing household wealth exposure to single asset price volatility, but this study finds that this dimension has relatively weak protective effects, primarily constrained by rural financial market development levels, with limited types of financial assets available to households (8). The debt constraint mechanism indicates that good debt management avoids households falling into excessive debt traps (15, 30), preventing debt burden from exacerbating economic hardship when facing medical expenditure shocks. Among the four dimensions, saving behavior and insurance awareness demonstrate the strongest protective effects, determined by the unique characteristics of health risk. Health risk simultaneously has high-frequency low-loss and low-frequency high-loss dual characteristics—the former includes common illnesses like colds with high occurrence frequency but relatively small single expenditures, while the latter includes major diseases like cancer with low occurrence probability but huge single expenditures. These dual characteristics require households to establish differentiated defense systems: savings primarily cope with high-frequency low-loss risks, while insurance primarily copes with low-frequency high-loss risks. The importance of savings is also reflected in the time lag of insurance reimbursement—the pay-first, reimburse-later mechanism means that even if reimbursement can ultimately be obtained, sufficient liquidity is needed at the time of medical treatment. The advantage of insurance lies in its leverage effect, converting small fixed premiums into large payouts for major illnesses, and commercial health insurance complements basic medical insurance, covering self-paid drugs and imported medical devices outside the basic insurance catalog. These findings suggest that financial capacity building should focus on savings and insurance dimensions, helping rural households establish defense systems matching the dual characteristics of health risk.

A significant substitutional relationship exists between health insurance and financial management behavior, and the strength of this substitution varies by household financial capacity. This finding directly addresses the core paradox raised in the introduction: why CHE incidence remains high despite 95% insurance coverage. This study indicates that the protective effect of health insurance is highly dependent on household financial foundations, with the same insurance coverage producing differentiated effects across households with different financial capacities (12, 18). Risk compensation mechanism and resource reallocation mechanism jointly explain this substitutional relationship. The risk compensation mechanism indicates that uninsured households face greater medical expenditure risk exposure, and this risk perception compels households to adopt more cautious financial management strategies, while insured households reduce the urgency of self-protection due to external security lowering risk exposure levels (21). The resource reallocation mechanism indicates that health insurance reduces the necessity of precautionary savings, thereby releasing household financial resources (17, 23). For low financial capacity households, funds released by insurance may flow toward basic living consumption to improve living standards; for high financial capacity households, funds released by insurance may be invested in human capital or productive investments. The substitution rate differences found in this study confirm this mechanism—low financial capacity households highly depend on insurance as primary security, while high financial capacity households primarily rely on self-protection, with insurance playing more of a supplementary role. This finding challenges the traditional assumption of simple additive external security and internal buffers, indicating that policy effects have conditional dependence (20). Health insurance policy should not adopt one-size-fits-all expansion strategies but should consider household financial capacity differences (24): for low financial capacity households, insurance is critical security, and policy focus should be on improving security levels; for high financial capacity households, incentive mechanisms such as individual insurance accounts and health points can encourage continued maintenance of good financial management habits, achieving synergistic development of insurance systems and household self-protection mechanisms. This differentiated strategy helps improve insurance resource allocation efficiency and provides a demand-side perspective for understanding the diminishing marginal effects of supply-side reforms.

Heterogeneity analysis findings confirm the law of diminishing marginal utility in economics and reveal complex interaction relationships among different dimensions of financial management behavior. The protective effect of financial management behavior is stronger among low-income households, western regions, and households headed by older adults, consistent with the law of diminishing marginal utility (3). For low-income households, each additional unit of financial management capacity may be critical for coping with medical expenditure shocks, while for high-income households the marginal impact is relatively limited. Western regions have relatively scarce medical resources and lower insurance reimbursement levels, making household financial reserves the primary reliance for coping with health shocks, with higher marginal value of financial management capacity (27). Households headed by older adults face triple pressures of high health risk, few income sources, and large medical expenditure proportions (25, 29), relying more on existing financial accumulations. This finding introduces scarcity mentality theory into health economics, proving that resource scarcity affects not only decision-making processes but also changes the marginal utility function of financial tools (28). More importantly, this study finds complementary and substitutional relationships among different dimensions of financial management behavior, a level less frequently examined in existing research (14, 16). Savings and debt management exhibit a substitutional relationship—when household savings are adequate, the marginal importance of debt management declines; savings and debt exhibit a complementary relationship—when household debt pressure is high, the buffering role of savings becomes more important; savings and insurance also have some substitutional relationship. These complex interaction relationships indicate that financial capacity building cannot adopt single-dimension intervention strategies but should implement differentiated interventions based on existing household financial structures. For high-debt households, the immediate priority is helping them establish saving habits, as marginal savings have extremely high value; for high-saving households, marginal resources should be invested in insurance rather than continuing to increase savings, as insurance’s leverage effect can obtain greater security at smaller cost; for households without commercial insurance, savings become the only line of defense, and accumulation should be prioritized. This systematic strategy based on comprehensive household financial profiles may be more effective than one-size-fits-all universal interventions.

This study has the following limitations. First, causal identification challenges. Although we employed instrumental variable methods and passed relevant tests, cross-sectional data limit exploration of dynamic causal relationships, and the sustained effects of financial behavior improvements and heterogeneity at different life cycle stages remain unclear (9). Individual traits such as time preference and risk preference are difficult to observe, and while instrumental variable methods partially alleviate this problem, concerns about omitted variable bias cannot be completely eliminated. In particular, cross-sectional data cannot fully distinguish whether households with higher savings are skilled financial managers or whether they accumulated savings following a previous health shock. While the instrumental variable approach partially addresses this concern, it cannot be completely resolved without longitudinal data tracking households before and after health events. Second, instrumental variable assumptions. The exclusion restriction—that village-level financial service accessibility affects CHE only through financial behavior—cannot be formally tested in cross-sectional data and remains a maintained assumption. Furthermore, while the larger 2SLS coefficient relative to the OLS baseline is consistent with attenuation bias from measurement error in the self-reported index, this interpretation cannot be formally verified with the available data. The baseline OLS results should therefore be regarded as a conservative lower bound of the true effect. Third, measurement precision limitations. The medical expenditure scope used in this study is relatively comprehensive, with CHE incidence higher than some literature (31, 32). Multiple threshold robustness tests indicate this difference does not affect core conclusions, but the distinction between necessary medical expenditure and improvement health consumption requires more detailed data. Notably, discrepancies may exist between objective CHE measurement and household subjective perceptions, and this inconsistency in subjective-objective assessment suggests future research needs to understand household medical economic burden more comprehensively (33). Fourth, external validity limitations. This study focuses on rural households; the mechanisms of financial management behavior in urban households and the applicability of insurance substitution effects in other developing countries require further testing.

Based on these limitations, the following research directions warrant exploration. Panel data or intervention experiments would help identify causal effects more rigorously. Future studies with panel data tracking households before and after health events would help verify the direction of causality and validate the instrumental variable assumptions adopted in this study. More detailed medical expenditure classification and alternative construction methods for financial management behavior indices may improve measurement precision. Urban–rural comparative studies and cross-national comparative studies would test the universality of research conclusions and provide evidence for understanding how institutional environments influence policy effects. Additionally, how to translate complementary-substitutional relationships among financial dimensions into operable precision intervention tools still requires further exploration through policy experiments.

## 5 Conclusion

This study constructed a financial management behavior index to reveal the internal mechanisms by which rural households cope with CHE risk. Based on large-sample empirical analysis of 17,156 rural households, findings indicate that financial management behavior significantly reduces CHE risk through three mechanisms—liquidity buffer, risk diversification, and debt constraint—and this causal relationship is more robust after controlling for endogeneity using instrumental variable methods. Among the four dimensions, saving behavior and insurance awareness constitute the core defense line against health shocks, with the former providing immediate payment capacity for high-frequency low-loss daily illnesses and the latter using leverage effects to cope with low-frequency high-loss major diseases, with their differentiated functions determined by the dual characteristics of health risk. More importantly, this study finds that rather than a simple additive relationship, health insurance and household financial management behavior exhibit significant substitution effects, with this substitution strength varying by household financial capacity. This finding challenges the traditional assumption of simple addition of external security and internal buffers, revealing policy intervention complexity. Heterogeneity analysis further indicates that the marginal value of financial management capacity is higher among resource-scarce groups, confirming the law of diminishing marginal utility, while complementary and substitutional relationships among different financial dimensions suggest limitations of single-dimension interventions. This study provides new theoretical perspectives and empirical evidence for understanding the role of household financial capacity in health risk management, offering reference for improving rural medical security systems and financial capacity building.

## Data Availability

Publicly available datasets were analyzed in this study. This data can be found at: the data used in this study can be accessed by application through the Survey and Research Center for China Household Finance at Southwestern University of Finance and Economics (https://chfs.swufe.edu.cn/).
